# Tau Pathology of Alzheimer Disease: Possible Role of Sleep Deprivation

**DOI:** 10.32598/bcn.9.5.307

**Published:** 2018-09-01

**Authors:** Nahid Ahmadian, Sajjad Hejazi, Javad Mahmoudi, Mahnaz Talebi

**Affiliations:** 1. Neurosciences Research Center, Tabriz University of Medical Sciences, Tabriz, Iran.; 2. Department of Molecular Medicine, Faculty of Advanced Medical Sciences, Tabriz University of Medical Sciences, Tabriz, Iran.; 3. Department of Anatomy, Faculty of Veterinary Medicine, Tabriz Branch, Islamic Azad University, Tabriz, Iran.

**Keywords:** Neurofilament proteins, Tau proteins, Amyloid beta-peptides, Amyloid beta-protein precursor, Alzheimer’s disease, Sleep disorder

## Abstract

Sleep deprivation is a common complaint in modern societies. Insufficient sleep has increased the risk of catching neurodegenerative diseases such as Alzheimer’s. Several studies have indicated that restricted sleep increases the level of deposition of β-amyloid and formation of neurofibrillary tangles, the major brain microstructural hallmarks for Alzheimer disease. The mechanisms by which sleep deprivation affects the pathology of Alzheimer disease has not yet been fully and definitively identified. However, risk factors like apolipoprotein E risk alleles, kinases and phosphatases dysregulation, reactive oxygen species, endoplasmic reticulum damages, glymphatic system dysfunctions and orexinergic system inefficacy have been identified as the most important factors which mediates between the two conditions. In this review, these factors are briefly discussed.

## Highlights

- Insufficient sleep increases the risk of catching some diseases such as Alzheimer Disease (AD).
- Losing just one night of sleep leads to an immediate increase in beta-amyloid, a protein in the brain associated with Alzheimer disease.

## Plain Language Summary

Sleep plays a crucial role in the continuity of cognitive sequences and executive functions. Sleep deprivation is a common complaint in modern societies. Insufficient sleep has increased the risk of catching some diseases such as Alzheimer Disease (AD). It is a type of dementia that causes problems with memory, thinking, and behavior. Symptoms usually develop slowly and get worse over time, becoming severe enough to interfere with daily task. AD is the most common cause of dementia, a general term for memory loss and other cognitive disabilities serious enough to interfere with daily life. Alzheimer disease accounts for 60% to 80% of dementia cases. AD worsens over time, where dementia symptoms gradually worsen over a number of years. In its early stages, memory loss is mild, but with late-stage, individuals lose the ability to carry on a conversation and respond to their environment. AD is the sixth leading cause of death in the United States. Those with Alzheimer live an average of eight years after their symptoms become noticeable to others, but survival can range from 4 to 20 years, depending on age and other health conditions. Losing just one night of sleep led to an immediate increase in beta-amyloid, a protein in the brain associated with Alzheimer disease.

## 1. Introduction

The biological and behavioral roles of sleep are not entirely elucidated. Studies show that sleep plays a crucial role in the continuity of cognitive sequences and executive functions (Cirelli, Shaw, Rechtschaffen, & Tononi, 1999; Wilckens, Woo, Kirk, Erickson, & Wheeler, 2014). Sleep Deprivation (SD) as a common phenomenon in the modern societies, endangers individuals‘ health both in acute and chronic states. The reduced sleep hours is related to alterations in living style, increase in night work hours, and late-night activities (Navara & Nelson, 2007). Studies show that people with insomnia or sleep disorders are at elevated risk for neuro-degenerative disorders such as Alzheimer Disease (AD) (Cedernaes et al., 2016). Moreover, SD are observed across moderate to severe AD and are worsened with the progression of the disease (Anderson & Bradley, 2013).

Tau is one of the most important Microtubule Associated Proteins (MAP) in the neurons. The balanced tau phosphorylation binds it to the microtubules, assembles the microtubules, and maintains the structure and stability of the neurons (Kadavath et al., 2015). However, hyperphosphorylation of tau results in its aggregation and formation of paired helical filamentous structures known as Neurofibrillary Tangles (NFTs) (Harrison & Owen, 2016). NFTs are among the main neurological hallmarks of AD (Cox, Davis, Mash, Metcalf, & Banack, 2016).

Evidence from animal models illustrates that changes in the sleep-wake cycle may elevate hyperphosphorylated Tau protein in the brain (Di Meco, Joshi, & Praticò, 2014; Rothman, Herdener, Frankola, Mughal, & Mattson, 2013). It is reported that two months of SD causes more than 50% elevation of the insoluble Tau in the brain (Nunomura et al., 2001). In addition, chronic SD increases extracellular amyloid, the main component of the amyloid plaques found in the brains of patients with Alzheimer (Sadigh-Eteghad et al., 2015) and sleep extension decreases plaques in animal models (Lim, Gerstner, & Holtzman, 2014).

It is indicated that Cerebrospinal Fluid (CSF) Amyloid-β (Aβ) levels predict amyloid plaque deposition, and sleep deprivation is associated with fluctuations in the level of *Aβ* in CSF and its deposition in the brain (Roh et al., 2012); even one night of sleep deprivation is proposed to be associated with a 6% decrease in CSF Aβ42 levels (Ooms et al., 2014). Therefore, in general, it seems that SD can affect Alzheimer pathology through intermediate mechanisms.

The current study aimed at reviewing the most significant factors mediating between SD and AD including Apolipoprotein E (*ApoE*) risk alleles, kinases, and phosphatases dysregulation, reactive oxygen species, endoplasmic reticulum damage, glymphatic system dysfunction, and orexinergic system inefficacy. Obviously, studying these factors in an integrated and concise way can be useful for researchers in the field of sleep and Alzheimer pathology.

## 2. Apolipoprotein E Risk Alleles

*ApoE*, as the main component of chylomicrons and Intermediate-Density Lipoproteins (IDLs), plays an essential role in the normal catabolism of lipoproteins (Huang & Mahley, 2014). *ApoE* transfers lipoproteins, fat-soluble vitamins, and cholesterol to the lymph vessels and then to the bloodstream. It is originally synthesized in the liver, but it is also abundant in the Central Nervous System (CNS) (Mahley, 2016). There are at least three variants (alleles) of the *APOE* gene, *ε2*, *ε3*, and *ε4* (Ryu, Atzmon, Barzilai, Raghavachari, & Suh, 2016).

*APOE ε4* genetic variant is known as the main genetic risk determinant of AD. The *ε4* allele also increases the risk for cerebral amyloid aggregation and age-related cognitive impairments (Liu, Kanekiyo, Xu, & Bu, 2013). It is believed that *APOE* risk variants can activate specific intracellular pathways by binding to surface receptors and peptides of the neurons, which ultimately leads to neurodegeneration and synaptic dysfunction (Giau, Bagyinszky, An, & Kim, 2015). Furthermore, pathological influence of *ApoE* on increasing cerebral *Aβ* deposition is demonstrated in several studies (Morris et al., 2010).

ApoE-lipoproteins, by removing the soluble *Aβ* from the extracellular matrix, can facilitate its uptake through LRP1, LDLR, and HSPG receptors. Additionally, the association between *ApoE*, 24S-hydroxycholesterol, and tau shows its direct involvement in generation of NFTs (Leoni, Solomon, & Kivipelto, 2010). On the other hand, the association of *APOE* with sleep disorders is also proven. For example, Kadotani et al. (2001) reported a significant relationship between sleep-disordered breathing and *APOE ε4* variant in the general population and Tisko et al. (2014) reported that Obstructive Sleep Apnea (OSA) is associated with *ε4* allele. In addition, Lim et al., (2013) proposed that specific *APOE ε4* genotypes may predispose individuals to sleep disruption and sleeping adequately inhibits the effect of *ApoE* on the formation of NFTs and progression of AD.

## 3. Kinases and Phosphatases Dysregulation

Protein kinases and phosphatases are two groups of enzymes that transfer phosphate to and from substrates such as tau protein (Cheng, Qi, Paudel, & Zhu, 2011; Nichol, Parachikova, & Cotman, 2007). Several protein kinases such as cyclic AMP-dependent Protein Kinase A (PKA), Calcium/calmodulin-dependent protein Kinase II (CaMKII), Glycogen Synthase Kinase-3β (*GSK-3β*), and Protein Phosphatases 2A (PP2A) have role in tau phosphorylation and de-phosphorylation (Shanavas & Papasozomenos, 2000).

PKA is one of the enzymes involved in sleep/wake regulation (Avila et al., 2012; Hellman, Hernandez, Park, & Abel, 2010). There is evidence that sleep deprivation causes PKA activation in the brain (Datta & Desarnaud, 2010; Graves et al., 2003) and its activation increases phosphorylation in multiple sites of tau (Ittner et al., 2016). CaMKII is a complex protein kinase and has an important role in synaptic plasticity and memory formation (Giese & Mizuno, 2013). Animal studies show that sleep deprivation remarkably dysregulates CaMKII-related phosphorylation (Cui et al., 2016). Furthermore, CaMKII is also a tau kinase and its dysregulation is associated with Alzheimer’s progression (Ghosh & Giese, 2015) and CaMKII inhibits tau-microtubule interaction by tau phosphorylation (Singh et al., 1996).

Evidence shows that *GSK-3β* is another enzyme that influences sleep-wake organization (Ahnaou & Drinkenburg, 2011; Albrecht, 2012; Hickie, Naismith, Robillard, Scott, & Hermens, 2013). This enzyme phosphorylates at least 36 residues of tau protein (Hanger et al., 2007). As a matter of fact, *GSK-3* activation is a critical step in brain aging and AD that triggers cascade of detrimental events such as NFT formation and neuronal death pathways (Takashima, 2006). On the other hand, an Extra-cellular signal-Regulated Kinase (ERK) is centrally involved in memory consolidation process (Kelly, Laroche, & Davis, 2003). Phosphorylation of ERK is a key step to inhibit its activity in response to synaptic stimuli (Grewal, York, & Stork, 1999). Guan, Peng and Fang (2004) demonstrated that SD impairs ERK phosphorylation process in the hippocampus and this leads to spatial memory impairments in rats. Future studies may clarify the role of this protein in the relationship between sleep disorders and AD in humans.

## 4. Reactive Oxygen Species

Reactive Oxygen Species (ROS) are a number of molecular oxygen-derived reactive molecules and free radicals produced as byproducts during the mitochondrial electron transport or through oxidoreductase enzymatic activities (Ray, Huang, & Tsuji, 2012). Recent works demonstrated that ROS have a role in cellular signaling cascades including apoptosis, cell cycle regulation, phagocytosis, enzyme activation, and gene expression. The imbalance of ROS and antioxidant capacity of cells lead to oxidative stress (Dixon & Stockwell, 2014). Generally, due to the higher metabolic rate and the low rate of regeneration, nerve cells are more susceptible to oxidative damage (Manoharan et al., 2016).

Studies show that inefficient antioxidant system and the excess of free radicals such as superoxide anion, hydrogen peroxide, and nitric oxide may be among the causes of the emergence of AD (Xie et al., 2002). Positive association between the amyloid plaque and 4-hydroxynonenal and malondialdehyde as the main lipid peroxidation markers proves this hypothesis to some extent (Massaad, 2011). Furthermore, it is demonstrated that iron accumulation in the brain of patients with AD is responsible for generating free radicals through the Fenton reaction (Zhao & Zhao, 2013). On the other hand, it is shown that SD can reduce the function of the antioxidant system of the cells. Ramanathan, Gulyani, Nienhuis and Siegel (2002) showed that prolonged sleep deprivation profoundly decreased superoxide dismutase antioxi-dative activity in the hippocampus and brainstem of rats.

Mathangi, Shyamala and Subhashini (2012) reported that paradoxical sleep deprivation was a potent oxidative stressor, which likely played a role in the behavioral changes of animal models. It is also shown in humans that SD can increase malondialdehyde (El-Helaly & Abu-Hashem, 2010). Therefore, SD increases the oxidative stress and may thus contribute to the etiology of AD.

## 5. Endoplasmic Reticulum Damages

The Endoplasmic Reticulum (ER) is involved in folding and trafficking of proteins, redox homeostasis, energy production, and apoptosis (Cao & Kaufman, 2014). Malfunctions of the ER may lead to a cell stress response, which can eventually trigger programmed cell death. Increasing evidence emphasizes on the role of ER in the development and progression of neurodegenerative diseases (Scheper & Hoozemans, 2015). Studies indicate that apoptosis generally occurs through two main pathways: the death receptor (extrinsic) and the mitochondrial (intrinsic) pathways (Elmore, 2007).

ER stress has a key role in the pathogenesis of AD. Inositol-Requiring kinase 1 (IRE1) initiates the ER stress pathway by triggering Apoptosis Signal-regulating Kinase 1 (ASK1), which in turn activates c-Jun N-terminal Kinase (JNK) signaling route (Okazawa & Estus, 2002). This cascade has the potential to trigger AD pathogenesis through dysregulation of Amyloid Precursor Protein (APP) processing and intracellular *Aβ* accumulation, activation of Activator Protein 1 (AP-1), a transcription factor that regulates inflammatory genes expression, and hyperphosphorylation of tau protein, and aggregation of neurofibrillary tangles (Viana, Nunes, & Rodrigues, 2012). Also, ER stress causes deposition of unfolded or misfolded proteins such as tau (Kanemoto & Wang, 2012; Naidoo, 2009). Prolonged presence of these toxic unfolded proteins triggers intrinsic apoptosis pathways (Fribley, Zhang, & Kaufman, 2009).

Degradation of tau decreased by 20% in ER stress due to decrease in the binding of tau to CHIP (carboxyl terminus of Hsc70-interacting protein), which delayed the degradation of tau through the ubiquitin-proteasome pathway. SD increases ER stress in brain tissue (Sakagami et al., 2013). It is proposed that REM SD elevates the level of noradrenaline in the brain (Ranjan, Biswas, & Mallick, 2010). Animal studies indicate that REM SD changes BAX and Bcl2 functions and initiate mitochondrial apoptosis pathway (Ranjan et al., 2010). Somarajan, Khanday and Mallick (2016) suggested that elevated noradrenaline acting on α1-adrenergic receptor causes mitochondrial damage, release of cytochrome c, and induction of apoptosis intrinsic pathway. Therefore, SD can be related to the Alzheimer’s pathology through induction of neuronal apoptosis.

## 6. Glymphatic System Dysfunctions

Iliff et al. (2012) discovered the dynamic characteristics of the glymphatic system in mice, using in vivo two-photon microscopy. By fluorescent labeling of CSF, they showed the rapid entry of CSF into the brain along the cortical and pial arteries, following the influx into the Virchow-Robin spaces through penetrating arterioles. In fact, the CSF enters the parenchyma through a definite periarterial pathway surrounded by perivascular astrocytic end-feet (Iliff et al., 2012). It can be concluded that glymphatic system plays a key role in feeding the neurons and purgation of the brain environment.

Glymphatic system works differently during sleep and awakening. It is hypothesized that during the sleep, the CSF flows more profusely and the elimination of toxic substances from neurons and intercellular spaces is greatly increased. When sleep is restricted, glymphatic system does not have enough time to fulfill its function; hence, toxins and misfolded proteins are accumulated, and the effects will appear in cognitive capabilities and executive functions (Eugene & Masiak, 2015). On the other hand, Weller et al. (2008) demonstrated that macroscopic glymphatic system-based clearance of interstitial metabolites may be of particular significance for neurodegenerative diseases such as AD, in which, the accumulation of protein aggregates is observed (Iliff et al., 2012).

In this regard, Iliff et al. (2012) found that *Aβ* was rapidly broken down and eliminated in the glymphatic system route. In other words, imbalance between *Aβ* production and clearance can result in accumulation of *Aβ* and emergence of AD. As a result, SD may be associated with a decrease in the ability of the glymphatic system and an increase in the accumulation of *Aβ* and Alzheimer etiology.

## 7. Orexinergic System Inefficacy

The orexins (hypocretins), as hypothalamic neuropeptides, play an important role in sleep-wake cycle regulation (Sakurai, Pandi-Perumal, & Monti, 2015). Abnormal levels of these neuropeptides in people with sleep disorders led to research into its role in sleep regulation (Ebrahim, Howard, Kopelman, Sharief, & Williams, 2002). In humans, orexinergic neurons are restricted to the dorsolateral hypothalamus, and project densely to various regions such as the Locus Coeruleus (LC), amygdala, suprachiasmatic nucleus, dorsal raphe nuclei, and cholinergic brainstem (Mieda & Sakurai, 2016).

Although the role of the orexinergic system in sleep regulation is not certainly known, however, it is believed that orexinergic projections modulate cholinergic and monoaminergic activities during the sleep cycle. Indeed, inputs from suprachiasmatic nucleus to the orexinergic system exhibit the dependence of this system function on the dark-light cycle (Hungs & Mignot, 2001). Studies show a significant reduction in the number of orexinergic neurons in human narcolepsy (Mieda & Sakurai, 2016).

Mehta, Khanday and Mallick (2015) measured orexin-A level in LC, cortex, posterior hypothalamus, hippocampus, and pedunculopontine areas after 96 hours REM SD in rats. They reported that following the REM SD, the orexin-A level significantly increased in LC, cortex, and posterior hypothalamus. Interestingly, they observed that after recovery, the level of orexin-A returned to its normal state.

On the other hand, it is demonstrated that increase in orexin due to chronic SD is involved in the pathogenesis of AD (Scammell, Matheson, Honda, Thannickal, & Siegel, 2012). Liguori et al. (2014) illustrated that patients with moderate-to-severe AD had higher levels of orexin and faced higher levels of nocturnal sleep disorders. In addition, it is proposed that orexin-A is associated with increased phosphorylated tau and this may be related to a reduction in the ratio of deep sleep (Osorio et al., 2016).

## 8. Result

Sleep and circadian rhythm disturbances possibly occur very early in the pathogenesis process of AD. Experimental models suggest that SD may increase the soluble *Aβ* levels and lead to chronic accumulation of Aβ, whereas improving time and quality of sleep has the opposite effect (Ju, Lucey, & Holtzman, 2013). Furthermore, it is proven that this relationship can be reversed, and the accumulation of *Aβ* may cause a sleep-wakefulness imbalance (Ju et al., 2013).

Using in vivo microdialysis in mice, Kang, Lim, Bateman, Lee and Smyth (2009) found that the amount of *Aβ* in brain interstitial fluid is correlated with wakefulness and increased during orexin infusion, but decreased with infusion of a orexin receptor antagonist. Roh et al. (2012) reported that disruption of the sleep-wake cycle can accelerate *Aβ* deposition in the mouse brain.

Spira et al. (2013) found that among older adults, shorter duration and poorer quality of sleep are associated with greater *Aβ* accumulation. Ooms et al. (2014) reported that evening and morning Aβ42 concentrations differed between the individuals with unrestricted sleep and those with SD. Ju et al. (2017) reported that specific disruption of slow wave activity may increase Aβ40 and poor sleep quality is associated with higher tau levels (Ju et al., 2017).

Di Meco et al. (2014) observed that SD decreases levels of postsynaptic density protein 95 and increases glial fibrillary acidic protein levels. Moreover, they investigated that total levels of the phosphorylated transcription factor cellular response element binding protein is significantly diminished in the brains of sleep-deprived mice compared with that of controls. They underlined the importance of SD as a chronic stressor in modulating biochemical processes involved in the development of AD.

In general, SD appears to increase the accumulation of *Aβ* and tau proteins, thus accelerating the formation of amyloid plaques and neurofibrillary tangles. On the other hand, increased accumulation of Aβs and tau proteins in turn disrupt the sleep-wake cycle, thus forming a defective cycle. It seems that increasing the duration and improving the quality of sleep can slow down the progression of the disease. However, given the extensive study of animals in this field (Mahmoudi, Ahmadian, Farajdokht, Majdi, & Erfani, 2017), most results are derived from animal studies and human clinical studies are required to confirm the obtained results.

## 9. Conclusions

The current study reviewed the most important factors in the relationship between SD and AD. It was discussed that *APOE ε4* allele could be associated with sleep disorders, tau phosphorylation, and *Aβ* deposition. SD can increase active oxygen species and as a result, damage mitochondria and induce apoptosis. SD reduces the activity of the glymphatic system, and consequently, reduces the capacity to remove Aβ. By activating kinases, SD may increase tau phosphorylation and cause the formation of neurofibrillary tangles, and all of the pathways in question, eventually lead to the emergence of AD through neuronal degeneration, *Aβ* deposition, and neurofibrillary tangles formation. Therefore, considering the important role of sleep in modulating the risk factors of AD, it may be possible to prevent the emergence of AD at aging by improving the quality of sleep and treating sleep disorders from early ages.

## Ethical Considerations

### Compliance with ethical guidelines

There is no ethical principle to be considered doing this research.

## References

[B1] Ahnaou A. Drinkenburg W. (2011). Disruption of glycogen synthase kinase-3-beta activity leads to abnormalities in physiological measures in mice. Behavioural Brain Research, 221(1), 246–52. [DOI:10.1016/j.bbr.2011.03.004] [PMID ] 21392539

[B2] Albrecht U. (2012). Circadian rhythms and sleep-the metabolic connection. Pflügers Archiv-European Journal of Physiology, 463(1), 23–30. [DOI:10.1007/s00424-011-0986-6] [PMID ] 21710201

[B3] Anderson K. N. Bradley A. J. (2013). Sleep disturbance in mental health problems and neurodegenerative disease. Nature and Science of Sleep, 5, 61–75. [DOI:10.2147/NSS.S34842] [PMID ] [PMCID ] PMC3674021 23761983

[B4] Avila J. León Espinosa G. García E. García Escudero V. Hernández F. DeFelipe J. (2012). Tau phosphorylation by GSK3 in different conditions. International Journal of Alzheimer’s Disease, 2012, Article ID: 578373. [DOI:10.1155/2012/578373] [PMID ] [PMCID ] PMC3362846 22675648

[B5] Cao S. S. Kaufman R. J. (2014). Endoplasmic reticulum stress and oxidative stress in cell fate decision and human disease. Antioxidants & Redox Signaling, 21(3), 396–413. [DOI:10.1089/ars.2014.5851] 24702237 PMC4076992

[B6] Cedernaes J. Osorio R. S. Varga A. W. Kam K. Schiöth H. B. Benedict C. (2016). Candidate mechanisms underlying the association between sleep-wake disruptions and Alzheimer’s disease. Sleep Medicine Reviews, 31, 102–11. [PMID ] [PMCID ] 26996255 10.1016/j.smrv.2016.02.002 PMC4981560

[B7] Cheng H. C. Qi R. Z. Paudel H. Zhu H. J. (2011). Regulation and function of protein kinases and phosphatases. Enzyme Research, 2011, 794089 [DOI:10.4061/2011/794089] 22195276 PMC3238372

[B8] Cirelli C. Shaw P. J. Rechtschaffen A. Tononi G. (1999). No evidence of brain cell degeneration after long-term sleep deprivation in rats. Brain Research, 840(1), 184–93. [DOI:10.1016/S0006-8993(99)01768-0] 10517970

[B9] Cox P. A. Davis D. A. Mash D. C. Metcalf J. S. Banack S. A. (2016). Dietary exposure to an environmental toxin triggers neurofibrillary tangles and amyloid deposits in the brain. Proceedings of the Royal Society B: Biological Sciences, 283(1823), 20152397 [DOI:10.1098/rspb.2015.2397] PMC4795023 26791617

[B10] Cui S. Y. Li S. J. Cui X. Y. Zhang X. Q. Yu B. Sheng Z. F. (2016). Phosphorylation of CaMKII in the rat dorsal raphe nucleus plays an important role in sleep-wake regulation. Journal of Neurochemistry, 136(3), 609–19. [DOI:10.1111/jnc.13431] [PMID ] 26558357

[B11] Datta S. Desarnaud F. (2010). Protein kinase A in the pedunculopontine tegmental nucleus of rat contributes to regulation of rapid eye movement sleep. The Journal of Neuroscience, 30(37), 12263–73. [DOI:10.1523/JNEUROSCI.1563-10.2010] [PMID ] [PMCID ] 20844122 PMC3327880

[B12] Di Meco A. Joshi Y. B. Praticò D. (2014). Sleep deprivation impairs memory, tau metabolism, and synaptic integrity of a mouse model of Alzheimer’s disease with plaques and tangles. Neurobiology of Aging, 35(8), 1813–20. [DOI:10.1016/j.neuurobiolaging.2014.02.011] 24629673

[B13] Dixon S. J. Stockwell B. R. (2014). The role of iron and reactive oxygen species in cell death. Nature Chemical Biology, 10(1), 9–17. [DOI:10.1038/nchembio.1416] [PMID ] 24346035

[B14] Ebrahim I. O. Howard R. S. Kopelman M. D. Sharief M. K. Williams A. J. (2002). The hypocretin/Orexin system. Journal of the Royal Society of Medicine, 95(5), 227–30. [DOI:10.1177/014107680209500503] [PMID ] [PMCID ] 11983761 PMC1279673

[B15] El Helaly M. Abu Hashem E. (2010). Oxidative stress, melatonin level, and sleep insufficiency among electronic equipment repairers. Indian Journal of Occupational and Environmental Medicine, 14(3), 66–70. [DOI:10.4103/0019-5278.75692] 21461157 PMC3062017

[B16] Elmore S. (2007). Apoptosis: A review of programmed cell death. Toxicologic Pathology, 35(4), 495–516. [DOI:10.1080/01926230701320337] 17562483 PMC2117903

[B17] Eugene A. R. Masiak J. (2015). The neuroprotective aspects of sleep. MEDtube Science, 3(1), 35–40. [PMID ] [PMCID ] 26594659 PMC4651462

[B18] Fribley A. Zhang K. Kaufman R. J. (2009). Regulation of apoptosis by the unfolded protein response. Methods in Molecular Biology, 559, 191–204. [DOI:10.1007/978-1-60327-017-5_14] 19609758 PMC3684430

[B19] Ghosh A. Giese K. P. (2015). Calcium/calmodulin-dependent kinase II and Alzheimer’s disease. Molecular Brain, 8(1), 78. [DOI:10.1186/s13041-015-0166-2] [PMID ] [PMCID ] 26603284 PMC4657223

[B20] Giau V. V. Bagyinszky E. An S. S. Kim S. Y. (2015). Role of apolipoprotein E in neurodegenerative diseases. Neuropsychiatric Disease and Treatment, 2015(11), 1723–37. [DOI:10.2147/NDT.S84266] PMC4509527 26213471

[B21] Giese K. P. Mizuno K. (2013). The roles of protein kinases in learning and memory. Learning & Memory, 20(10), 540–52. [DOI:10.1101/lm.028449.112] [PMID ] 24042850

[B22] Graves L. A. Hellman K. Veasey S. Blendy J. A. Pack A. I. Abel T. (2003). Genetic evidence for a role of CREB in sustained cortical arousal. Journal of Neurophysiology, 90(2), 1152–9. [DOI:10.1152/jn.00882.2002] [PMID ] 12711709

[B23] Grewal S. S. York R. D. Stork P. J. (1999). Extracellular-signal-regulated kinase signalling in neurons. Current Opinion in Neurobiology, 9(5), 544–53. [DOI:10.1016/S0959-4388(99)00010-0] 10508738

[B24] Guan Z. Peng X. Fang J. (2004). Sleep deprivation impairs spatial memory and decreases extracellular signal-regulated kinase phosphorylation in the hippocampus. Brain Research, 1018(1), 38–47. [DOI:10.1016/j.brainres.2004.05.032] 15262203

[B25] Hanger D. P. Byers H. L. Wray S. Leung K. Y. Saxton M. J. Seereeram A. (2007). Novel phosphorylation sites in tau from Alzheimer brain support a role for casein kinase 1 in disease pathogenesis. Journal of Biological Chemistry, 282(32), 23645–54. [DOI:10.1074/jbc.M703269200] [PMID ] 17562708

[B26] Harrison J. R. Owen M. J. (2016). Alzheimer’s disease: The amyloid hypothesis on trial. The British Journal of Psychiatry, 208(1), 1–3. 26729836 10.1192/bjp.bp.115.167569

[B27] Hellman K. Hernandez P. Park A. Abel T. (2010). Genetic evidence for a role for protein kinase A in the maintenance of sleep and thalamocortical oscillations. Sleep, 33(1), 19–28. [DOI:10.1093/sleep/33.1.19] [PMID ] [PMCID ] 20120617 PMC2802244

[B28] Hickie I. B. Naismith S. L. Robillard R. Scott E. M. Hermens D. F. (2013). Manipulating the sleep-wake cycle and circadian rhythms to improve clinical management of major depression. BMC Medicine, 11, 79. [DOI:10.1186/1741-7015-11-79] [PMID ] [PMCID ] 23521808 PMC3760618

[B29] Hoon Roh J. Yafei H. Bero A. W. Kasten T. (2012). Disruption of the sleep-wake cycle and diurnal fluctuation of β-Amyloid in mice with Alzheimer’s disease pathology. Science Translational Medicine, 4(150), 150ra122. [DOI:10.1126/scitranslmed.3004291] PMC3654377 22956200

[B30] Huang Y. Mahley R. W. (2014). Apolipoprotein E: Structure and function in lipid metabolism, neurobiology, and Alzheimer’s diseases. Neurobiology of Disease, 72, 3–12. [DOI:10.1016/j.nbd.2014.08.025] [PMID ] [PMCID ] 25173806 PMC4253862

[B31] Hungs M. Mignot E. (2001). Hypocretin/orexin, sleep and narcolepsy. BioEssays, 23(5), 397–408. [DOI:10.1002/bies.1058] 11340621

[B32] Iliff J. J. Wang M. Liao Y. Plogg B. A. Peng W. Gundersen G. A. (2012). A Paravascular Pathway Facilitates CSF flow through the brain parenchyma and the clearance of interstitial solutes, including Amyloid β. Science Translational Medicine, 4(147), 147ra111. [DOI:10.1126/scitranslmed.3003748] PMC3551275 22896675

[B33] Ittner A. Chua S. W. Bertz J. Volkerling A. van der Hoven J. Gladbach A. (2016). Site-specific phosphorylation of tau inhibits amyloid-β toxicity in Alzheimer’s mice. Science, 354(6314), 904–8. [DOI:10.1126/science.aah6205] [PMID ] 27856911

[B34] Ju Y. E. S. Lucey B. P. Holtzman D. M. (2013). Sleep and Alzheimer disease pathology-a bidirectional relationship. Nature Reviews Neurology, 10(2), 115–9. [DOI:10.1038/nrneuerol.2013.269] 24366271 PMC3979317

[B35] Ju Y. S. Ooms S. J. Sutphen C. Macauley S. L. Zangrilli M. A. Jerome G. (2017). Slow wave sleep disruption increases cerebrospinal fluid amyloid-beta levels. Brain, 140(8), 2104–11. [DOI:10.1093/brain/awx148] 28899014 PMC5790144

[B36] Kadavath H. Hofele R. V. Biernat J. Kumar S. Tepper K. Urlaub H. (2015). Tau stabilizes microtubules by binding at the interface between tubulin heterodimers. Proceedings of the National Academy of Sciences, 112(24), 7501–6. [DOI:10.1073/pnas.1504081112] [PMID ] [PMCID ] PMC4475932 26034266

[B37] Kadotani H. Kadotani T. Young T. Peppard P. E. Finn L. Colrain I. M. (2001). Association between apolipoprotein E∈4 and sleep-disordered breathing in adults. JAMA, 285(22), 2888–90. [DOI:10.1001/jama.285.22.2888] [PMID ] 11401610

[B38] Kanemoto S. Wang H. (2012). Roles of endoplasmic reticulum stress in neurodegenerative diseases. Translational Medicine, 2012, 2:e108.

[B39] Kang J. E. Lim M. M. Bateman R. J. Lee J. J. Smyth L. P. (2009). Amyloid-β dynamics are regulated by Orexin and the sleep-wake cycle. Science, 326(5955), 1005–7. [DOI:10.1126/scicence.1180962] 19779148 PMC2789838

[B40] Kelly Á. Laroche S. Davis S. (2003). Activation of mitogen-activated protein kinase/Extracellular signal-regulated kinase in hippocampal circuitry is required for consolidation and reconsolidation of recognition memory. Journal of Neuroscience, 23(12), 5354–60. [DOI:10.1523/JNEUROSCI.23-12-05354.2003] [PMID ] 12832561 PMC6741214

[B41] Leoni V. Solomon A. Kivipelto M. (2010). Links between ApoE, brain cholesterol metabolism, tau and amyloid β-peptide in patients with cognitive impairment. Biochemical Society Transactions, 38(4), 1021–5. [DOI:10.1042/BST0381021] 20658997

[B42] Liguori C. Romigi A. Nuccetelli M. Zannino S. Sancesario G. Martorana A. (2014). Orexinergic system dysregulation, sleep impairment, and cognitive decline in Alzheimer disease. JAMA Neurology, 71(12), 1498–505. [DOI:10.1001/jaamaneurol.2014.2510] [PMID ] 25322206

[B43] Lim A. S. P. Yu L. Kowgier M. Schneider J. A. Buchman A. S. Bennett D. A. (2013). Sleep modifies the relation of APOE to the risk of Alzheimer disease and neurofibrillary tangle pathology. JAMA Neurology, 70(12), 1544–51. [DOI:10.1001/jamaneurol.2013.4215] 24145819 PMC3859706

[B44] Lim M. M. Gerstner J. R. Holtzman D. M. (2014). The sleep-wake cycle and Alzheimer’s disease: What do we know?. Neurodegenerative Disease Management, 4(5), 351–362. [DOI:10.2217/nmt.14.33] 25405649 PMC4257134

[B45] Liu C. C. Kanekiyo T. Xu H. Bu G. (2013). Apolipoprotein E and Alzheimer disease: Risk, mechanisms, and therapy. Nature Reviews. Neurology, 9(2), 106–18. [DOI:10.1038/nrneuurol.2012.263] 23296339 PMC3726719

[B46] Mahley R. W. (2016). Central nervous system lipoproteins: ApoE and regulation of cholesterol metabolism. Arteriosclerosis, Thrombosis, and Vascular Biology, 36(7), 1305–15. [DOI:10.1161/ATVBAHA.116.307023] PMC4942259 27174096

[B47] Mahmoudi J. Ahmadian N. Farajdokht F. Majdi A. Erfani M. (2017). A protocol for conventional sleep deprivation methods in rats. Journal of Experimental and Clinical Neuroscience, 4(1), 1–4. [DOI:10.13183/jecns.v4i1.61]

[B48] Manoharan S. Guillemin G. J. Abiramasundari R. S. Essa M. M. Akbar M. Akbar M. D. (2016). The role of reactive oxygen species in the pathogenesis of Alzheimer’s disease, Parkinson’s disease, and Huntington’s disease: A mini review. Oxidative Medicine and Cellular Longevity, 2016, 8590578 [DOI:10.1155/2016/8590578] 28116038 PMC5223034

[B49] Massaad C. A. (2011). Neuronal and vascular oxidative stress in Alzheimer’s disease. Current Neuropharmacology, 9(4), 662–73. [DOI:10.2174/157015911798376244] 22654724 PMC3263460

[B50] Mathangi D. C. Shyamala R. Subhashini A. S. (2012). Effect of REM sleep deprivation on the antioxidant status in the brain of Wistar rats. Annals of Neurosciences, 19(4), 161–4. [DOI:10.5214/ans.0972.7531.190405] 25205991 PMC4117056

[B51] Mehta R. Khanday M. A. Mallick B. N. (2015). REM sleep loss associated changes in orexin-A levels in discrete brain areas in rats. Neuroscience Letters, 590, 62–7. [DOI:10.1016/j.neulet.2015.01.067] 25637698

[B52] Mieda M. Sakurai T. (2016). Orexin (hypocretin) and narcolepsy. In Goswami M. Thorpy M.J. Pandi Perumal S.R. (Eds), Narcolepsy (pp. 11–23). Berlin: Springer [DOI:10.1007/978-3-319-23739-8_2]

[B53] Morris J. C. Roe C. M. Xiong C. Fagan A. M. Goate A. M. Holtzman D. M. (2010). APOE predicts amyloid-beta but not tau Alzheimer pathology in cognitively normal aging. Annals of Neurology, 67(1), 122–31. [DOI:10.1002/ana.21843] [PMID ] [PMCID ] 20186853 PMC2830375

[B54] Naidoo N. (2009). Cellular stress/the unfolded protein response: Relevance to sleep and sleep disorders. Sleep Medicine Reviews, 13(3), 195–204. [DOI:10.1016/j.smrv.2009.01.001] [PMID ] [PMCID ] 19329340 PMC2964262

[B55] Navara K. J. Nelson R. J. (2007). The dark side of light at night: Physiological, epidemiological, and ecological consequences. Journal of Pineal Research, 43(3), 215–24. [DOI:10.1111/j.1600-079X.2007.00473.x] [PMID ] 17803517

[B56] Nichol K. E. Parachikova A. I. Cotman C. W. (2007). Three weeks of running wheel exposure improves cognitive performance in the aged Tg2576 mouse. Behavioural Brain Research, 184(2), 124–32. [DOI:10.1016/j.bbr.2007.06.027] [PMID ] [PMCID ] 17698211 PMC2215770

[B57] Nunomura A. Perry G. Aliev G. Hirai K. Takeda A. Balraj E. K. (2001). Oxidative damage is the earliest event in Alzheimer disease. Journal of Neuropathology & Experimental Neurology, 60(8), 759–67. [DOI:10.1093/jnen/60.8.759] 11487050

[B58] Okazawa H. Estus S. (2002). The JNK/c-Jun cascade and Alzheimer’s disease. American Journal of Alzheimer’s Disease & Other Dementias, 17(2), 79–88. [DOI:10.1177/153331750201700209] PMC10833950 11954673

[B59] Ooms S. Overeem S. Besse K. Rikkert M. Verbeek M. Claassen J. R. (2014). Effect of 1 night of total sleep deprivation on cerebrospinal fluid β-amyloid 42 in healthy middle-aged men: A randomized clinical trial. JAMA Neurology, 71(8), 971–77. [DOI:10.1001/jamaneurol.2014.1173] 24887018

[B60] Osorio R. S. Ducca E. L. Wohlleber M. E. Tanzi E. B. Gumb T. Twumasi A. (2016). Orexin-A is associated with increases in cerebrospinal fluid phosphorylated-tau in cognitively normal elderly subjects. Sleep, 39(6), 1253–60. [DOI:10.5665/sleep.5846] 26951396 PMC4863214

[B61] Ramanathan L. Gulyani S. Nienhuis R. Siegel J. M. (2002). Sleep deprivation decreases superoxide dismutase activity in rat hippocampus and brainstem. Neuroreport, 13(11), 1387–90. [DOI:10.1097/00001756-200208070-00007] [PMID ] 12167758 PMC8802885

[B62] Ranjan A. Biswas S. Mallick B. N. (2010). Cytomorphometric changes in the dorsal raphe neurons after rapid eye movement sleep deprivation are mediated by noradrenalin in rats. Behavioral and Brain Functions, 6, 62 [DOI:10.1186/1744-9081-6-62] 20964843 PMC2984478

[B63] Ray P. D. Huang B. W. Tsuji Y. (2012). Reactive Oxygen Species (ROS) homeostasis and redox regulation in cellular signaling. Cellular Signalling, 24(5), 981–90. [DOI:10.1016/j.cellsig.2012.01.008] 22286106 PMC3454471

[B64] Roh J. H. Huang Y. Bero A. W. Kasten T. Stewart F. R. Bateman R. J. (2012). Disruption of the sleep-wake cycle and diurnal fluctuation of beta-amyloid in mice with Alzheimer’s disease pathology. Science Translational Medicine, 4(150), 150ra122. [DOI:10.1126/scitranslmed.3004291] PMC3654377 22956200

[B65] Rothman S. M. Herdener N. Frankola K. A. Mughal M. R. Mattson M. P. (2013). Chronic mild sleep restriction accentuates contextual memory impairments, and accumulations of cortical Aβ and pTau in a mouse model of Alzheimer’s disease. Brain Research, 1529, 200–8. [DOI:10.1016/j.brainnres.2013.07.010] [PMID ] [PMCID ] 23856323 PMC3779872

[B66] Ryu S. Atzmon G. Barzilai N. Raghavachari N. Suh Y. (2016). Genetic landscape of APOE in human longevity revealed by high-throughput sequencing. Mechanisms of Ageing and Development, 155, 7–9. [DOI:10.1016/j.mad.2016.02.010] [PMID ] [PMCID ] 26930295 PMC4818712

[B67] Sadigh-Eteghad S. Sabermarouf B. Majdi A. Talebi M. Farhoudi M. Mahmoudi J. (2015). Amyloid-beta: A crucial factor in Alzheimer’s disease. Medical Principles and Practice, 24(1), 1–10. [DOI:10.1159/000369101] PMC5588216 25471398

[B68] Sakagami Y. Kudo T. Tanimukai H. Kanayama D. Omi T. Horiguchi K. (2013). Involvement of endoplasmic reticulum stress in tauopathy. Biochemical and Biophysical Research Communications, 430(2), 500–4. [DOI:10.1016/j.bbrc.2012.12.007] [PMID ] 23237806

[B69] Sakurai T. Pandi-Perumal S. R. Monti J. M. (2015). Orexin and sleep. Berlin, Springer [DOI:10.1007/978-3-319-23078-8]

[B70] Scammell T. Matheson J. Honda M. Thannickal T. Siegel J. (2012). Coexistence of narcolepsy and Alzheimer’s disease. Neurobiology of Aging, 33(7), 1318–9. [DOI:10.1016/j.neurobioolaging.2010.12.008] [PMID ] 21257235 PMC8720268

[B71] Scheper W. Hoozemans J. J. (2015). The unfolded protein response in neurodegenerative diseases: A neuropathological perspective. Acta Neuropathologica, 130(3), 315–31. [DOI:10.1007/s00401-015-1462-8] 26210990 PMC4541706

[B72] Shanavas A. Papasozomenos S. C. (2000). τ kinases in the rat heat shock model: Possible implications for Alzheimer disease. Proceedings of the National Academy of Sciences, 97(26), 14139–44. [DOI:10.1073/pnas.97.26.14139] [PMID ] [PMCID ] PMC18884 11121021

[B73] Singh T. J. Wang J. Z. Novak M. Kontzekova E. Grundke Iqbal I. Iqbal K. (1996). Calcium/Calmodulin-dependent protein kinase II phosphorylates tau at Ser-262 but only partially inhibits its binding to microtubules. FEBS Letters, 387(2–3), 145–8. [DOI:10.1016/0014-5793(96)00485-1] 8674537

[B74] Somarajan B. I. Khanday M. A. Mallick B. N. (2016). Rapid eye movement sleep deprivation induces neuronal apoptosis by noradrenaline acting on alpha1 adrenoceptor and by triggering mitochondrial intrinsic pathway. Frontiers in Neurology, 7, 25 [DOI:10.3389/fneur.2016.00025] 27014180 PMC4779900

[B75] Spira A. P. Gamaldo A. A. An Y. Wu M.N. Simonsick E.M. Bilgel M. (2013). Self-reported sleep and β-amyloid deposition in community-dwelling older adults. JAMA Neurology, 70(12), 1537–43. [DOI:10.1001/jamaneurol.2013.4258] 24145859 PMC3918480

[B76] Takashima A. (2006). GSK-3 is essential in the pathogenesis of Alzheimer’s disease. Journal of Alzheimer’s Disease, 9(3 Suppl), 309–17. [DOI:10.3233/JAD-2006-9S335] 16914869

[B77] Tisko R. Sopkova Z. Habalova V. Dorkova Z. Slaba E. Javorsky M. (2014). Effects of apolipoprotein E genotype on serum lipids in obstructive sleep apnoea. European Respiratory Journal, 43(4), 1097–05. [DOI:10.1183/09031936.00098513] 24232699

[B78] Viana R. J. Nunes A. F. Rodrigues C. M. (2012). Endoplasmic reticulum enrollment in Alzheimer’s disease. Molecular Neurobiology, 46(2), 522–34. [DOI:10.1007/s12035-012-8301-x] 22815194

[B79] Wilckens K. A. Woo S. G. Kirk A. R. Erickson K. I. Wheeler M. E. (2014). Role of sleep continuity and total sleep time in executive function across the adult lifespan. Psychology and Aging, 29(3), 658. [DOI:10.1037/a0037234] [PMID ] [PMCID ] 25244484 PMC4369772

[B80] Xie Z. Wei M. Morgan T. E. Fabrizio P. Han D. Finch C. E. (2002). Peroxynitrite mediates neurotoxicity of amyloid beta-peptide1-42- and lipopolysaccharide-activated microglia. Journal of Neuroscience, 22(9), 3484–92. [DOI:10.1523/JNEUROSCI.22-09-03484.2002] [PMID ] 11978825 PMC6758387

[B81] Zhao Y. Zhao B. (2013). Oxidative stress and the pathogenesis of Alzheimer’s disease. Oxidative Medicine and Cellular Longevity, 2013, 316523. [DOI:10.1155/2013/316523] [PMID ] [PMCID ] 23983897 PMC3745981

